# Retrospective analysis of hospital discharge records for cases of trichinellosis does not allow evaluation of disease burden in Italy

**DOI:** 10.1051/parasite/2019043

**Published:** 2019-07-16

**Authors:** Edoardo Pozio, Alessandra Ludovisi, Patrizio Pezzotti, Fabrizio Bruschi, Maria Ángeles Gómez-Morales

**Affiliations:** 1 Department of Infectious Diseases, Istituto Superiore di Sanità viale Regina Elena 299 00161 Rome Italy; 2 Department of Translational Research, N.T.M.S. Università di Pisa via Roma 55 56126 Pisa Italy

**Keywords:** *Trichinella*, Hospital discharge records, Epidemiology, Italy, Prevalence, Incidence

## Abstract

Human trichinellosis is a disease caused by nematode worms of the genus *Trichinella*. In Italy, as well as in most other European countries, notification of *Trichinella* infections in humans is mandatory; however, no information is available on the number of cases occurring annually. The aim of the present study was to retrospectively evaluate the burden of trichinellosis in Italy from 2005 to 2016. Hospital discharge records (HDRs) showing the code for trichinellosis (124) were registered and screened. Results were then compared with yearly reports issued by the Italian National Reference Laboratory for *Trichinella* (NRLT), with reports from the European Centre for Disease Prevention and Control (ECDC), and with literature data. A total of 102 HDRs revealed that the 124 code was erroneously reported in 72 (70.6%) records. Out of the 30 (29.4%) records with a correct diagnosis of trichinellosis, nine cases were reported by HDRs only, 21 cases were documented by both HDRs and the NRLT, whereas the NRLT documented 100 additional cases. In the studied period, the average yearly incidence was 0.01 cases per 100,000 inhabitants. This study highlights the limitations of using HDRs to obtain a clear picture of the prevalence and incidence of trichinellosis in Italy. These findings demonstrate the need to intensify the surveillance system for trichinellosis through the development of an Italian registry. This would allow the identification of patients with severe infections and pauci-symptomatic patients, and would avoid the need for clinical analyses and unnecessary treatments, reducing the resulting economic burden on the Italian National Health Service.

## Abstract (fr)

La trichinellose humaine est une maladie causée par les nématodes du genre *Trichinella*. En Italie, ainsi que dans la plupart des autres pays Européens, la notification des infections à *Trichinella* chez l’homme est obligatoire, mais aucune information n’est disponible sur le nombre de cas survenant chaque année. La présente étude visait à évaluer de manière rétrospective la charge de la trichinellose en Italie de 2005 à 2016. Les dossiers de sortie d’hôpital (DSH) indiquant le code de la trichinellose (124) ont été enregistrés et examinés. Les résultats ont ensuite été comparés aux rapports annuels du Laboratoire national de référence italien pour *Trichinella* (NRLT), du Centre européen de prévention et de contrôle des maladies (ECDC) et aux données de la littérature. L’analyse des 102 dossiers de sortie a montré que le code 124 avait été utilisé par erreur pour 72 dossiers (70,6 %). Parmi les 30 dossiers (29,4 %) avec un diagnostic correct de trichinellose, neuf cas avaient été identifiés seulement par le DSH et 21 cas à la fois par le DSH et par le NRLT. Sur la même période, le NRLT avait documenté 100 cas supplémentaires. Au cours de la période de l’étude, l’incidence annuelle moyenne était de 0,01 cas pour 100 000 habitants. Cette étude a mis en évidence les limites de l’utilisation des DSH pour obtenir une image fidèle de la prévalence et de l’incidence de la trichinellose en Italie. Cette étude identifie la nécessité d’intensifier le système de surveillance de la trichinellose par la mise en place d’un registre italien. Cela permettrait d’identifier les patients pauci-symptomatiques ou atteints d’infections sévères, et éviterait le recours à des analyses cliniques et à des traitements inutiles, ce qui réduirait la charge économique qui en résulte pour le Service National de Santé italien.

## Introduction

Trichinellosis is a cosmopolitan infectious disease caused by nematode parasites of the genus *Trichinella*. Human infection occurs through the ingestion of muscle-encysted larvae in raw or semi-raw meat and meat products derived from pigs, horses and wild omnivorous and/or carnivorous animals [31]. A systematic review of the scientific literature on worldwide cases of human trichinellosis published between 1986 and 2009 showed that the majority of infections (56,912; 86.47%) were recorded in Europe [35]. Trichinellosis can be severe when the infecting dose is high, particularly in the elderly, who may develop complications such as myocarditis or encephalitis possibly leading to death [9, 10, 33].

The number of trichinellosis cases within the European Union (EU) increased during the 1970s and until the end of the 1990s due to the growing prevalence of *Trichinella* spp. infections in domestic pigs of Eastern European origin (Bulgaria, Poland and Romania), and the occurrence of large outbreaks caused by the consumption of horse meat imported into France and Italy from Eastern Europe and other regions (Canada, Mexico and the United States) [4, 36, 37]. The prevalence of trichinellosis in Europe slowly decreased following the implementation of control programs in pig husbandry and the disappearance of *Trichinella* infections caused by horse meat consumption, following the introduction of controls at the abattoir [11, 12].

In Italy, trichinellosis was documented for the first time in 1887; only four sporadic cases and an outbreak with two deaths were recorded in Central and Northern Italy up to 1930 [8]. From 1933 to 1946, four large outbreaks involving 209 patients with 22 deaths occurred in Sicily through the consumption of infected pig meat [38]. After World War II to the year 2000, 1383 cases in the context of 22 outbreaks, and about 70 single cases, were documented to have been caused by the consumption of infected pig, horse or wild boar meat or meat-derived products [8, 39]. From 2001 to 2004, 12 infections, two of which were acquired abroad (Croatia), were documented in the context of three outbreaks [41] (Pozio E., unpublished data). Although trichinellosis is a notifiable disease in Italy, the real prevalence and incidence is unknown due to the absence of pathognomonic signs and symptoms, and the lack of highly sensitive and specific laboratory tests available on the market.

The aim of the present study was to retrospectively evaluate the burden of trichinellosis in Italy from 2005 to 2016, based on hospital discharge records (HDRs). The results of HDRs were compared with yearly reports issued by the Italian National Reference Laboratory for *Trichinella* (NRLT) and those of the European Centre for Disease Prevention and Control (ECDC), as well as with literature data.

## Materials and methods

The Italian hospital information system (Ihis), established in 1995, routinely collects data on all public and private hospital discharges and covers 100% of hospital admissions in Italy [2]. Ihis systematically collects both demographic and clinical information, including primary diagnosis and up to five subsequent diagnoses, primary and secondary diagnostic/therapeutic procedures, and length of hospital stay. Diagnoses and procedures are coded using the “International Classification of Diseases, 9th Revision, Clinical Modification” (ICD-9-CM) [32]. The data are sent by hospitals to the Regional Health Authority that is responsible for data quality verification before communicating the information to the Italian Ministry of Health.

The HDRs contain an anonymous individual code for tracking the patient’s hospital admissions, discharges and readmissions. Each anonymous individual code identifies one patient. Data from HDRs for the period from 2005 to 2016 were used. Hospital discharges were selected on the basis of the ICD-9-CM code 124 for trichinellosis, when indicated in a primary or secondary diagnosis. Information retrieved included hospital code, name and address of the hospital, patient code, gender, region, place of residence, trichinellosis diagnosis if reported as the primary or secondary diagnosis, and hospital admission and discharge dates.

In January 2018, details of the retrospective project were emailed to the 54 hospitals registered within the health system of the Italian Ministry of Health as having patient admissions with the ICD-9-CM code 124. Hospitals were invited to submit a hard or electronic copy of anonymized patient records for which individual codes were available to the Department of Infectious Diseases at the *Istituto Superiore di Sanità* (Italian National Institute of Health). The deadline for record forwarding was set as April 15, 2018. In addition, if patient sera were still available, hospitals were invited to ship samples to the NRLT for confirmation of positive serology. This project was approved by the *Istituto Superiore di Sanità* ethics committee (Prot. PRE-18/18 of January 11, 2018).

The screening of medical records was based on the ECDC case definition [13] and the algorithm proposed by Dupouy-Camet and Bruschi [9]. The number of trichinellosis cases was then compared with trichinellosis infections documented by the NRLT, ECDC and the international literature for the same time period.

Sera forwarded by hospitals to the NRLT were tested with a validated ELISA using excretory/secretory (ES) antigens as a first screening assay, and ELISA-positive sera were confirmed by a validated Western blot using ES antigens [29, 30].

## Results

During the period between 2005 and 2016, a total of 102 HDRs showing the trichinellosis 124 code were registered. All hospitals provided the requested medical records in electronic or hardcopy forms. Within this group, diagnosis of trichinellosis was reported either as the principal diagnosis or as the secondary diagnosis (up to the fifth level of secondary diagnosis). The screening of 102 records revealed that the 124 code was erroneously reported in 72 (70.6%) records provided by 42 hospitals as detailed in Table 1. Four serum samples, which had tested positive for *Trichinella* by commercial kits at hospital laboratories and sent together with the HDRs to our laboratory, tested negative by the validated serological tests used at the NRLT.

**Table 1:** Features of hospital discharge records (HDRs) with the 124 code (for trichinellosis) downloaded from the Italian Ministry of Health website for the period 2005–2016.

HDR features	No. (%)
Records with the 124 code erroneously reported due to:	72 (70.6)
– Typing errors	43 (42.1)
– Use of a new HDR code for the hospital readmission of trichinellosis cases at follow-up	8 (7.8)
– Inadequate diagnostic tools^a^	5 (4.9)
– Presence of only eosinophilia	5 (4.9)
– Absence of clarity between the genus name *Trichuris* and *Trichinella*	4 (3.9)
– Serum sample(s) tested by unreliable commercial kit	4 (3.9)
– Absence of clarity between trichilemmoma and trichinellosis	2 (1.9)
– Absence of clarity between trichiasis and trichinosis	1 (0.9)
Records correctly reporting the 124 code	30 (29.4)
Total records	102 (100)

a Copromicroscopic test of fecal samples (three patients, one of whom with *Trichuris trichiura* eggs); colonoscopy with biopsy (one patient) and without biopsy (one patient).

Out of 30 (29.4%) records with a correct diagnosis of trichinellosis on the basis of the ECDC case definition, five patients had acquired the infection in Romania and developed the disease in Italy from 2006 and 2013, and one patient acquired trichinellosis through the consumption of pork imported into Italy in 2009 from Romania. Four patients had acquired trichinellosis by the consumption of wild boar meat in different regions of the country. From 2005 to 2007, 19 patients had acquired trichinellosis through the consumption of pork in the Sardinia region. A trichinellosis infection was documented in Ancona hospital (Marche region, Central Italy) in 2005, but no information on the source of infection was available (Table 2).

**Table 2:** Human trichinellosis cases documented in Italy from 2005 to 2016 according to hospital discharge records (HDRs), surveillance by the National Reference Laboratory for *Trichinella* (NRLT), and ECDC reports.

Year	Locality (region)	No. of cases according to HDRs	No. of cases according to NRLT (No. of hospitalized patients)	Source of infection (etiological agent)^a^	No. of cases per year according to ECDC^b^
2005	Ancona (Marche)	1	n.d.^c^	Unknown	Italy did not provide any information
	Avezzano (Abruzzo)	2	n.d.	Hunted wild boar (Tb)	
	Cagliari (Sardinia)^d^	4	5 (4)	Free ranging pig (Tb)	
	Nuoro (Sardinia)^d^	8	8 (8)	Free ranging pig (Tb)	
	Mantua (Lombardy)^e^	n.d.	7 (7)	Horse meat imported from Belgium (Tb)	
2006	Verona (Veneto)	1	n.d.	Pork consumption in Romania (Ts)	Italy did not provide any information
	Nuoro (Sardinia)^f^	6^g^	6 (6)	Free ranging pig (Tb)	
	Lombardy	n.d.	3 (3)	Imported pork from Romania (Ts)	
2007	Nuoro (Sardinia)^g^	1	1 (1)	Free ranging pig (Tb)	1
2008	Tortona (Piedmont)	1	n.d.	Pork consumption in Romania (Ts)	Italy did not provide any information
	Verona (Veneto)^h^	n.d.	4 (4)	Pork consumption in Romania (Ts)	
2009	Cuneo (Piedmont)^i^	1	6 (1)	Hunted wild boar (Tb)	1
	Verona (Veneto)	1	n.d.	Imported pork from Romania (Ts)	
2010	Monza (Lombardy)	1	n.d.	Pork consumption in Romania	0
2011	Nuoro (Sardinia)^j^	n.d.	6 (6)	Free ranging pig (Tb)	6
2012	Valle del Serchio (Tuscany)^k^	1	34 (6)	Hunted wild boar (Tb)	33
	Treviso (Friuli Venezia Giulia)	1	n.d.	Pork consumption in Romania (Ts)	
2013	Torino (Piedmont)	1	n.d.	pork consumption in Romania (Ts)	Italy did not provide any information
2014	Latronico (Basilicata)	n.d.	4 (1)	Hunted wild boar (Tb)	4
2015	Genova (Liguria)	n.d.	30 (4)	Suspected hunted wild boar (Tp)	36^l^
2016	Manfredonia (Apulia)^m^	n.d.	5 (1)	Hunted wild boar (Tb)	5
2016	Pescara (Abruzzo)^n^	n.d.	1 (1)	Pork consumption in Romania (Ts)	
2016	Mattinata (Apulia)	n.d.	1 (1)	Hunted wild boar (Tb)	
Total		30	121 (54)		86^k^

a Ts = *Trichinella spiralis*; Tb = *Trichinella britovi*; Tp = *Trichinella pseudospiralis*.

b [14–25].

c n.d. = not documented.

d [42].

e [37].

f [40].

g Eight additional HDRs refer to the follow-up of the same patients.

h [1].

i [43].

j [3].

k [27].

l Six persons with positive serology did not meet the ECDC case definition of trichinellosis.

m [44].

n [28].

Discrepancies were observed between data provided by HDRs, the NRLT and ECDC (Table 2). From 2005 to 2016, there were 130 reported cases of trichinellosis in Italy, of which 9 were documented by HDRs only, 21 by both HDRs and the NRLT, and 100 by the NRLT only. Of the 130 documented infections, the Italian Ministry of Health provided only 86 (66.1%) cases to the ECDC (Table 2). The nine cases documented only by HDRs included a small outbreak involving two patients, and seven single cases of which six were acquired abroad (Romania), and one acquired in Italy. The examination of the NRLT reports and/or published data for the period between 2005 and 2016 showed that 54 (39.7%) patients were hospitalized, whereas only 30 (54.5%) hospitalized patients were documented by HDRs (Table 2). During the investigated 12-year period, the average annual incidence was 0.01 cases per 100,000 inhabitants (range 0.001–0.06).

Information on the etiological agents of trichinellosis cases was available in 98.4% of cases. *Trichinella britovi* was documented in 65.4% of infections, *Trichinella pseudospiralis* in 23.0%, and *Trichinella spiralis* in 10.0% (Table 2). All the *T. spiralis*-infected patients had eaten meat from animals reared abroad whose meat was either consumed abroad or was imported illegally (pork) into Italy, where the meat was subsequently consumed raw.

In the period under study, the main source of infection was meat and meat-derived products of illegally hunted wild boar (63.0%), followed by free-ranging pigs (20.0%), pork consumption in Romania (7.7%), horse meat imported from abroad (5.4%), and pork imported from Romania (3.0%) (Table 2). No information was available on the source of infection of a single case (0.8%), which was diagnosed in Ancona (Marche region) in 2005.

## Discussion

The examination of patient records with the ICD-9-CM code 124, issued in a 12-year period (2005–2016), showed that in most cases (*n* = 72; 70.6%) the code had been erroneously assigned as a result of typing errors (*n* = 43, 42.1%), the use of a new patient code (*n* = 8; 7.8%) at the time of readmission of trichinellosis cases for follow-up, the use of inappropriate diagnostic methods (e.g., detection of “parasites” in fecal samples or colonoscopy) (*n* = 5, 4.9%), poor knowledge of case definition (*n* = 10, 9.8%), absence of clarity with regards to worm terminology (*Trichuris* versus *Trichinella*) (*n* = 4, 3.9%), the use of invalidated serological kits (*n* = 4, 3.9%), and misspelling of the disease during reporting (*n* = 3, 2.9%) (Table 1).

Excluding typing errors and the use of different codes for the same patient, 21 (20.6%) inaccurate diagnoses were probably related to the lack of expertise regarding human trichinellosis seen at the hospital level. This may be due to both the rarity of the disease and the lack of emphasis on including diseases caused by helminths within the curricula of the majority of Italian Medical Faculties [6].

Within the EU, the notification of human *Trichinella* is mandatory in all countries, except for Belgium, France, and the United Kingdom which possess voluntary surveillance systems. In contrast, no surveillance system for trichinellosis exists in Denmark [25]. From 2005 to 2016, the trend for trichinellosis in the EU was greatly influenced by several outbreaks, with peaks often occurring through January and February. According to the ECDC, the annual number of trichinellosis cases documented in the EU decreased from 867 in 2007 to 224 in 2017 [26]. This reduction in human infections of about 75% is mainly due to improvements in pig rearing practices in Eastern European countries (Bulgaria, Croatia and Romania), increased control measures at the slaughterhouse, and the education of consumers, farmers and butchers on methods of transmission and prevention.

The occurrence in Italy during the period 2005–2016 of 14 cases of trichinellosis (10.7% of the total number of cases documented in Italy in the investigated period) with infection links originating from Romania is not surprising. Within the EU, Romania continues to represent the main focus of *Trichinella* infections among domestic pigs [26]. Seven infections occurred in Mantua (Lombardy region, Northern Italy) through the consumption of horse meat imported from abroad [37]. The 26 cases documented in two hospitals in Sardinia refer to four outbreaks and a single case, which occurred in 2005, 2006, 2011 and a single case in 2007. All these infections were caused by the consumption of raw meat from illegally slaughtered free-ranging pigs [3, 34, 40, 42]. The case of hospitalization in Lucca province (Tuscany region, Central Italy) refers to the index patient of an outbreak of trichinellosis, which involved 34 patients who had consumed raw sausages made with meat from a hunted wild boar in 2012 [27]. Another case was the index patient of an outbreak that involved six individuals who had consumed raw meat of a hunted wild boar in Cuneo province (Piedmont region, Northern Italy) in 2009 [43].

No HDRs were available for six outbreaks of trichinellosis involving 59 patients, 26 of whom were hospitalized and two single cases for which the NRLT performed serological tests and epidemiological investigations from 2005 to 2016 (Table 2).

**Figure 1:**
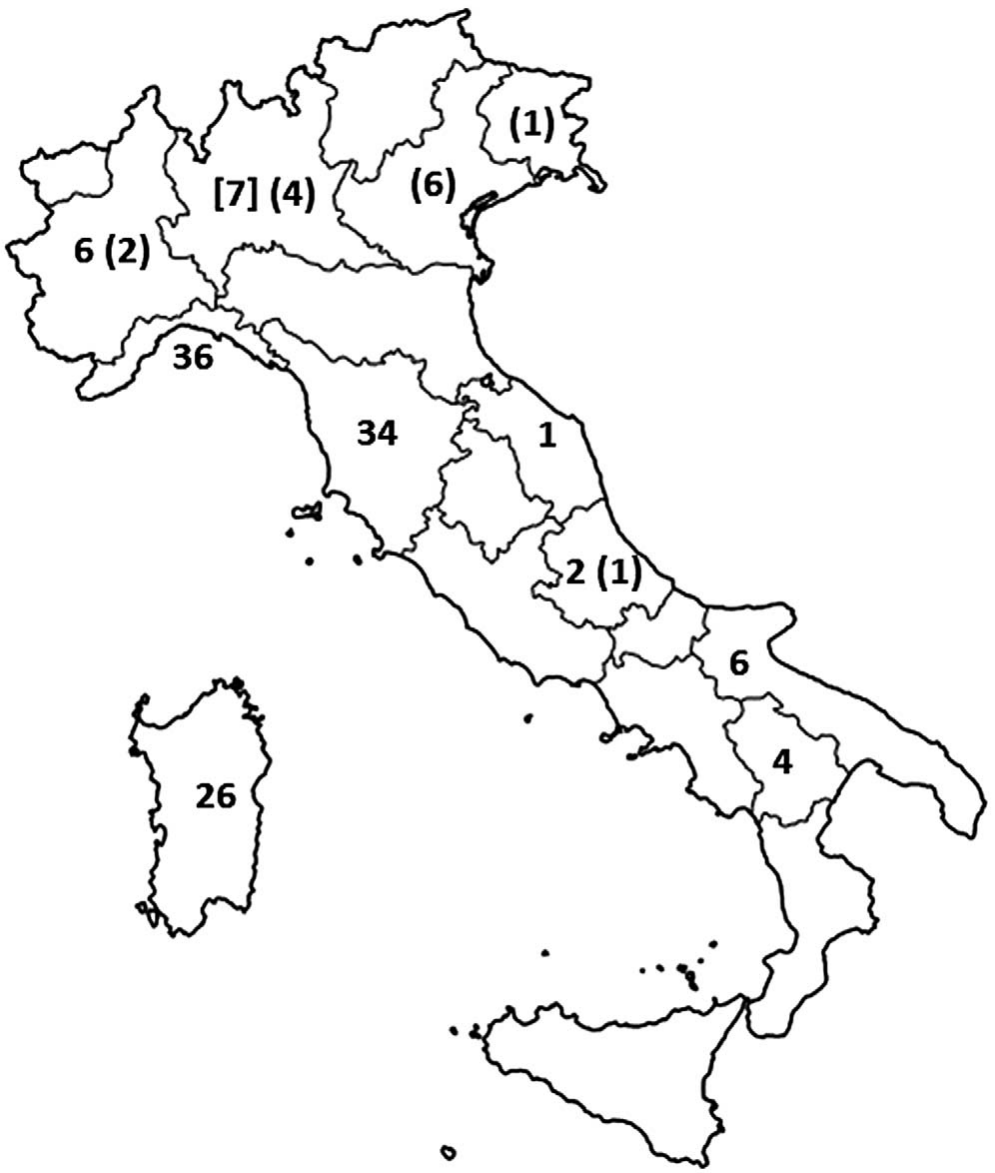
Distribution per region of trichinellosis cases documented in Italy from 2005 to 2016. Numbers in brackets are infections acquired in Romania and developed in Italy, or acquired in Italy through the consumption of illegally imported pork from Romania. The number in square brackets refers to trichinellosis cases acquired through the consumption of horse meat imported from Belgium.

From 2005 to 2016, trichinellosis infections were documented in 11 out of 20 Italian regions, without substantial differences between northern, central and southern Italy (Fig. 1). However, it is noteworthy that in 13 of the 14 trichinellosis cases, infection was acquired abroad (Romania) and illness developed in Italy, or they were caused through the consumption in Italy of pork from Romania illegally imported into Northern Italy, where about 50% of Romanian immigrants present in Italy live [45].

This study shows the limitation of the use of HDRs to obtain information on trichinellosis in Italy, as was already shown for other nematode infections, namely echinococcosis and anisakidosis [5, 7]. Since patients with severe or moderately severe trichinellosis quite frequently develop cardiovascular and neurological symptoms, as well as other complications, they could be discharged with an HDR code not indicating trichinellosis but other diseases. The number (*n* = 33, 25.4%) of inpatients with confirmed trichinellosis documented by the NRLT, but not by HDRs, strongly supports this hypothesis. In addition, during the course of a trichinellosis outbreak, only a low percentage of infected patients are hospitalized and this number is related not only to the severity of the infection, but also to the expertise of the physician and to different policies for hospitalization in different regions of Italy. It therefore follows that some patients access hospitals as outpatients for blood collection and treatment, but are neither reported nor registered by HDRs.

In addition, the retrospective retesting of serum samples, tested positive for *Trichinella* infection at the hospital laboratory, but not confirmed at the NRLT, emphasizes the low specificity of commercial kits, primarily due to the use of crude antigens that cross-react with other parasitic and non-parasitic antigens [29, 30].

The reduction in prevalence of parasitic diseases in industrialized countries in the 20th century resulted in the loss of diagnostic expertise among physicians, microbiologists and biologists. Additionally, meaningful investments in the development of new diagnostic tools for parasitic diseases remain scarce. A plethora of commercial kits for the diagnosis of parasitic infections, including trichinellosis, is on the market; however, most of these kits have not been validated by an independent body, resulting in poor diagnostic power. Furthermore, in Italy, due to financial constraints, some diagnostic laboratories purchase the cheapest commercial kits without taking into consideration their performance.

The results of the present study show that the surveillance system of the NRLT lost only 6.6% of trichinellosis infections that occurred in the studied period, whereas HDRs do not provide useful epidemiological information for this disease. This study suggests the need to develop an Italian registry on trichinellosis and to collect and disseminate information on the clinical and laboratory patterns, the long incubation time, and seroconversion times of this zoonotic disease. This information will support physicians for a diagnosis of choice and public health services to carry out epidemiological investigations during the course of trichinellosis outbreaks. The identification not only of patients with severe infections, but also of pauci-symptomatic patients, will eliminate the need for clinical analyses and unnecessary treatments, and thus help reduce the resulting economic burden on the Italian National Health Service.
